# The Challenges and Recommendations of Accessing to Affected Population for Humanitarian Assistance: A Narrative Review

**DOI:** 10.5539/gjhs.v7n3p111

**Published:** 2014-11-17

**Authors:** Shandiz Moslehi, Farin Fatemi, Mohammad Mahboubi, Hossein Mozafarsaadati, Shirzad Karami

**Affiliations:** 1 Department of Disaster Public Health, School of public Health, Tehran University of Medical Sciences, Tehran, Iran; 2 Department of Disaster and Emergency Health, National Institute of Health Research, Tehran University of Medical Sciences, Tehran, Iran; 3 Abadan School of Medical Sciences, Abadan, Iran; 4 Department of Epidemiology and Biostatistics, School of Public Health, Tehran University of Medical Science, Tehran, Iran; 5 Health Care Administration, Imam Hossein Hospital-Kermanshah City, Kermanshah, Iran

**Keywords:** access, humanitarian assistance, affected population, negotiation

## Abstract

**Objective:**

Access to affected people pays an important role in United Nation Organization for Coordination and Humanitarian Affairs (OCHA). The aim of this article is to identify the main obstacles of humanitarian access and the humanitarian organization responses to these obstacles and finally suggest some recommendations and strategies.

**Methods:**

In this narrative study the researchers searched in different databases. This study focused on the data from five countries in the following areas: access challenges and constraints to affected population and response strategies selected for operations in the affected countries by humanitarian organizations.

**Results:**

Three main issues were studied: security threats, bureaucratic restrictions and indirect constraint, which each of them divided to three subcategories. Finally, nine related subcategories emerged from this analysis.

**Conclusion:**

Most of these constraints relate to political issues. Changes in policy structures, negotiations and advocacy can be recommended to solve most of the problems in access issues.

## 1. Introduction

Humanitarian principles play an important role in crisis and disasters. The main core of humanitarian principles is neutrality, impartiality and independence (UN, 2013). For care giving we need the acceptance approaches and providing a basis on which warring parties may accept humanitarian action in situations of crisis and disasters. Some recent articles and publications also document the increasing challenges involved in maintaining humanitarian principles, and the implications for humanitarian access (Schreter, 2013).

Access is a fundamental precondition to effective humanitarian action (Mahbobi, Ojaghi, Rezayi & Khorasani, 2013) and is the main point in humanitarian assistance. As the Wikipedia defines the access, “accessibility” is the degree to which a product, device, service, or environment is available to as many people as possible. Accessibility can be viewed as the “ability to access” and benefit from some system or entity. The concept often focuses on people with disabilities or special needs (Wikipedia, 2013). In another dictionary access is defined as following: “the ability, right, or permission to approach, enter, speak with, or use” (Dictionary Reference, 2013). From our perspective, the concept of humanitarian access concerns both the ability of humanitarian organizations to reach populations affected by crises and the ability of affected populations to access humanitarian services (UNOCHA, 2013), not the just access of people to care in general. The access of affected population to care is an important issue, but it is not the focus of this study.

Full access is essential to perform operations, transfer goods and personnel where they are needed, implement distributions, provide health services and other activities (Bennett, 2012). We define full humanitarian access as the ability of all members of humanitarian organizations and donors to visit project implementation sites at the time of their choosing to provide humanitarian needs-based assistance and protection to people affected by crises, in line with the principles of humanity, neutrality, impartiality and independence (UN, 2013).

OCHA has a vital role in facilitating and coordinating humanitarian actors’ efforts to establish and maintain access and to overcome factors that inhibit access (Bennett, 2012), but in the field there may be some obstacles. These obstacles also affect the ability of affected populations to have full access to humanitarian aid. Humanitarian assistance means the provision of commodities and materials required to prevent and alleviate human suffering and do not include the provision of weapons, weapons systems, or other equipment, vehicles, or material which can be used in wars cause to harm or death of human.

Governments may limit access through different restrictions, criminal or terrorist gangs pose various security threats against humanitarian staff and other obstacles may make it very difficult to reach populations suffering from the effects of conflicts or disasters. These and other constraints make it difficult to deliver aid with neutrality, impartiality and independence (ECHO, 2012). So, due to these obstacles and problems to access the population, the aim of this article is to identify the main obstacles of humanitarian access and the humanitarian organization responses to these obstacles and finally suggest some recommendations and strategies by a narrative review.

## 2. Methods

This paper has been written using narrative review. The study team conducted a review of relevant literature according to the research question in both bibliographic and citation databases, some electronic humanitarian organization’s websites, electronic reports and documents which would exist in the internet. The search strategy was the same in the all searched materials. The “MeSH” terms used for searching were “humanitarian access”, “affected population”, constraint, emergencies, disasters in the title/abstract or keywords of articles and the other contents of searched materials.

Most data in the literature review came from Yemen, Somali, Sudan, Pakistan and Afghanistan in the last decade. The study focused on the five mentioned countries using the following criteria: the access challenges and constraints to affected population and the response strategies were selected for operations in the affected countries by humanitarian organizations. Finally, practical considerations and recommendation for accessing to affected population were taken into account in this study.

## 3. Results

On the basis of literature review from the papers, reports and documents the challenges and constraints for accessing to affected population in countries were investigated. This narrative review shows the obstacles to access affected population lie in the wide range of entry and movement restrictions, interference in the implementation of humanitarian activities until active hostilities and violence against humanitarian personnel and facilities (Bennett, 2012; ECHO, 2012). Different response policies have been designed against the obligatory obstacles on providing humanitarian aid to a needy population by active humanitarian organizations which were mentioned in the following table.

**Table 1:** Review of Access Obstacles & Humanitarian Organization Response

Access Obstacles	Humanitarian Organization Response
Not getting visa to humanitarian personnel from affected country.	Undertake humanitarian negotiations with the host country.
Movement restrictions of agencies, personnel or goods in major urban centers of affected country.	Undertake the humanitarian negotiations with the host country.
Significant level of violence in destination countries against humanitarian personnel & facilities.	Withdrew ongoing humanitarian assistance temporarily. Developing a joint inter agency response plan with a coordinated approach. Support NGO security bodies in providing training.
Diversion of food aid by armed groups	Making efforts to contact armed group
Extreme security threats such as kidnapping humanitarian aid workers by criminal gangs or suicide bomber attacks.	Making efforts to contact criminal gangs. Withdrew ongoing humanitarian assistance temporarily.
Restrictions imposed by governments for humanitarian aid workers.	Negotiations with government and limiting humanitarian assistance.
Attempts by parties to armed conflict to block access intentionally.	Withdrew the humanitarian assistance temporarily.

According to the above table, the researchers tried to categorize different access obstacles to affected population. Three main categories, security threats, bureaucratic restrictions and indirect constraint were found to be as the key access constraints which each of them divided to three subcategories. Finally, nine related subcategories emerged from this analysis.

**Figure 1:**
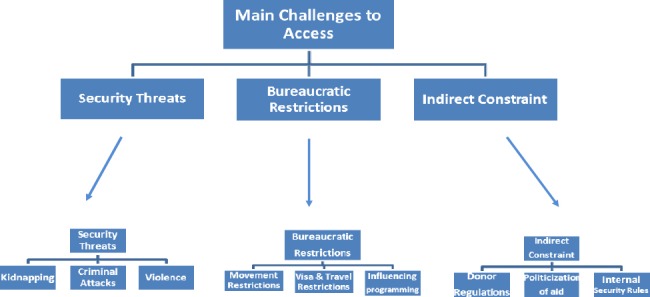
Key access challenges

## 4. Discussion

Experience shows that access is a major challenge in providing humanitarian aid to affected populations by a wide array of agencies and organizations, and will not require a significant military operation. Moreover, the interference of military power in humanitarian aid might interfere with the neutrality and impartiality of humanitarian principles, particularly in the countries engaged in civil war. In nearly all of reviewed cases about lack of access to affected population, negotiation is the first step of response which has been carried out by humanitarian agencies. At present due to increasing conflicts, there is an increasing need for accessing affected populations via humanitarian negotiation (International review of the Red Cross, 2011).

When the negotiation process is conducted in a more systematic and professional route, the results can lead to more consensuses with belligerents. The ability and skills of negotiators to engage the belligerents in a dialogue on humanitarian issues have determined the scope of humanitarian assistance and the degree of protection for large numbers of people (Reindorp & Wiles, 2001).

Humanitarian negotiation cannot be undertaken in the same way as political or commercial negotiations. This implies that predictability, trust, personal contact, cultural sensitivity and other deference should be considered in humanitarian negotiation. On several occasions (e.g. Iraq, Afghanistan and Sudan), the UN and others have responded to such access limitations by imposing conditional terms to the provision of humanitarian assistance or in Sierra Leone, the inter-agency consensus agreed to such military escorts in cases of extreme security risk (ECHO, 2012).

Making efforts to contact with armed group is another solution to have a better access to the victims. These efforts can not have a single model to mention in this article, but it could have some elements which should be considered. First, the armed group should be analyzed. The goal, ideologies, means of fighting, structure and essential character of armed groups should be studied. Identifying the leader and commander, the ideology which is lied under the war, the society organizations which support the armed group, sponsors, and the capacity of the armed group is important items in the first step. Understanding the lack of resources or the strengths of the armed group can help us to have a better negotiation. Second, there is a very important point which should be considered before intervention. This is fraternization problem. In this situation the mediator may be too close to the armed group. The third, is the analyzing the third party. The third party may increase the strengths or weaknesses of the groups. Having a weak mediator can cause some other problems. So, it is important to find a powerful and trained mediator. The last item which we should consider is the policies.

Withdraw ongoing humanitarian assistance temporarily is another humanitarian assistance organization strategies. Because the security of the humanitarian workers is important, in some situations they prefer to avoid assistance for a short time. Despite the fact that all the humanitarian assistance missions operate under explicit guidelines to remain politically neutral, aid workers are frequently the targets of violent attacks. So, understanding the security situation and determinants of violence against aid workers is important. As the other articles mentioned, in 2011, 308 aid workers were killed, kidnapped or wounded which is the highest number yet recorded (OCHA, 2012). Most of these attacks where took place in Afghanistan, Somalia, South Sudan, Pakistan. The number of workers was killed, kidnapped or wounded in 2008 was 260 (Stoddard, Harmer, & DiDomenico, 2009). As the results of the studies show, Afghanistan was the country with the highest number of attacks on aid workers in 2011 (OCHA, 2012). Mainly state authorities are responsible for the security of their citizens and other persons passing through the territory and also we have some regulations for aid worker security as follow: Convention on the Safety of United Nations and Associated Personnel (1994), Security Council Presidential Statement on the Protection of UN Personnel in Conflict Zones (2000), Security Council Resolution 1502 (2003), General Assembly Resolution 59/211 on the safety and security of humanitarian personnel and the protection of UN personnel (2004), and Optional Protocol to the Convention on the Safety of United Nations and Associated Personnel 60/581 (2005). But the result of insecurity is weak government. So, humanitarian organizations should improve situational awareness, risk assessment, mitigation measures and active acceptance strategies.

### 4.1 Limitation

In this narrative review only articles in the English language were searched and reviewed for the study. Also access to full text of several articles was not possible due to sanctions and economic difficulties.

## 5. Conclusion

Access to deliver assistance to affected populations is a major concern for humanitarian agencies and organizations today. As this review shows, access constraints have a long history in humanitarianism assistance. Most of these constraints relate to political issues. Many of humanitarian organizations have developed policies to deal with more common access constraints especially bureaucratic restrictions from host governments. Dealing with security threats needs more complex strategies and may be require a neutral third party for interference and to satisfy the belligerents in negotiation sessions. For removing indirect constraints against humanitarian assistance and donors, reconsideration of existing rules and considering retribution for outlaw counties is necessary. Experiences in most cases shows that military action for accessing to affected population contradicts the humanity, impartiality and neutrality principles which are mentioned in humanitarian law and the UN charter.

However, this narrative review suggest the following recommendations for overcoming the access constraints:

- Ministry of Planning and International Cooperation should explore ways to streamline the process for INGO registration, humanitarian project approval and etc.
- Governments should consider relaxing the information requirements for bureaucratic restrictions such as visa applications for temporary support staff or getting permission to movement in the affected area.
- Humanitarian agencies should increase communications awareness campaigns, particularly among displaced populations, vulnerable communities and among children living in conflict zones.
- Humanitarian actors should maintain humanitarian negotiations with relevant non–state actors in the affected country.
- Humanitarian agencies and organizations should continue to advocate to the belligerents and government.
- INGOs should ensure accurate and comprehensive reporting to UN humanitarian agencies and the Security Advisory Office of incidents which impact humanitarian access in affected countries.
